# Atypical radial plaques emanating from the umbilicus

**DOI:** 10.1016/j.jdcr.2024.11.027

**Published:** 2024-11-30

**Authors:** Helana Ghali, Meredith Thomley, Brandon Cardon, Wei-Shen Chen, Ann Lin

**Affiliations:** a University of South Florida, Morsani College of Medicine, Tampa, Florida; b Department of Dermatology and Cutaneous Surgery, University of South Florida, Tampa, Florida

**Keywords:** cutaneous metastasis, gastric cancer, signet ring cell adenocarcinoma, skin biopsy, umbilicus

## Case presentation

**Fig 1:**
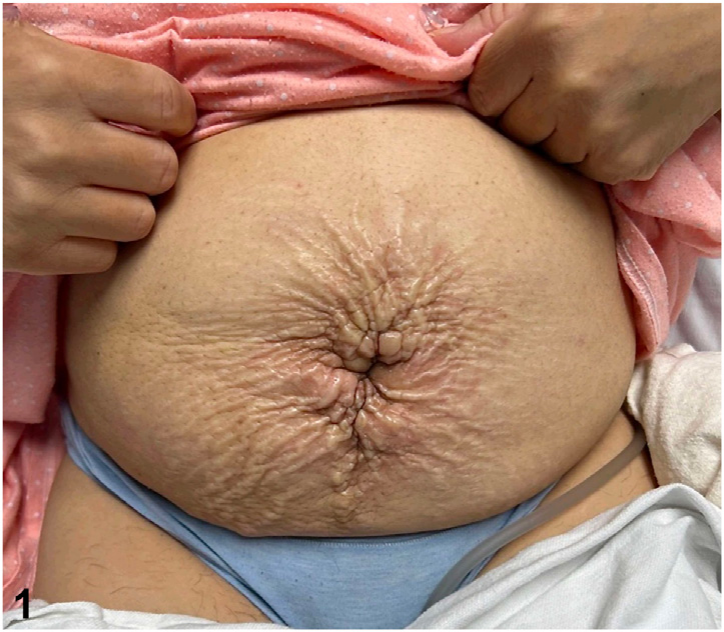

**Fig 2:**
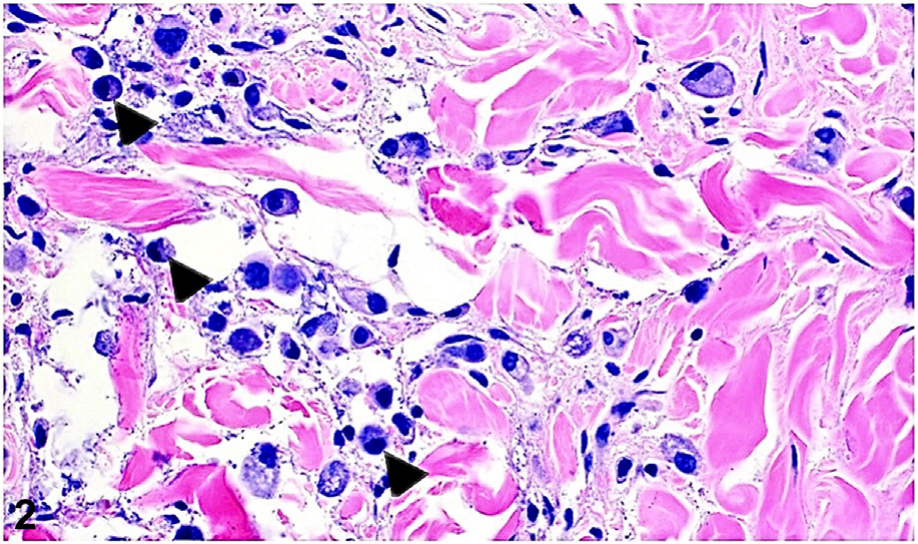

**Fig 3:**
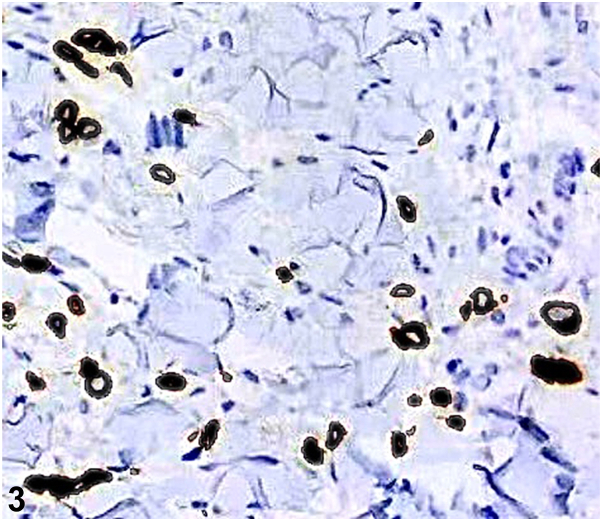

**Fig 4:**
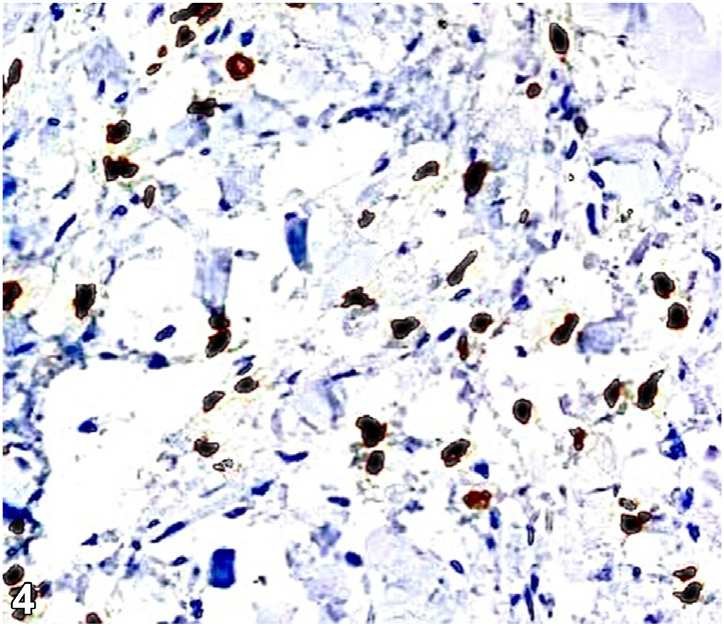

A 48-year-old Hispanic female presented with acute prandial epigastric pain, nausea, vomiting, abdominal distention, and a 40-pound weight loss over 6 months. Abdominal skin exam revealed diffuse thickening and atypical radial plaques around the umbilicus (Fig 1). The abdomen was also indurated and tender to palpation. A 4 mm punch biopsy revealed discohesive cells with eccentrically placed irregular nuclei and abundant intracytoplasmic mucin (Fig 2). Immunohistochemistry showed positivity for cytokeratin (CK)20, caudal type homeobox 2, and focal CK7 (Figs 3 and 4) and negativity for special AT-rich sequence binding protein 2, thyroid transcription factor, GATA binding protein, and paired box 8. Serology revealed elevated carcinoembyronic antigen (21.8 ng/mL) and cancer antigen (CA)-125 (151 kU/L) with normal CA-19-9 (<2.1 U/mL).

**Question 1: What is the most likely diagnosis?**

A. Metastatic colorectal cancer
B. Metastatic gastric signet ring cell adenocarcinoma
C. Ovarian cancer with peritoneal dissemination
D. Pancreatic cancer with liver metastasis
E. Primary peritoneal mesothelioma

**Answers:**

A. Metastatic colorectal cancer – Incorrect. Special AT-rich sequence binding protein 2 is identified as a relatively specific marker for both primary and metastatic signet ring cell adenocarcinomas of lower gastrointestinal tract origin. Histopathological examination would also reveal well-formed ductal structures infiltrating the dermis.
B. Metastatic gastric signet ring cell adenocarcinoma – Correct. This diagnosis is supported by the clinical presentation and characteristic histopathological findings of signet ring cells which include discohesive cells containing intracytoplasmic mucin with eccentrically positioned nuclei (Fig 2). This gives the tumor its distinctive histological appearance, often described as resembling a “signet ring.” Prior immunohistochemical studies of gastric signet ring cell adenocarcinoma have showed positivity for CK20, caudal type homeobox transcription factor, and CK7 all of which indicate a gastrointestinal origin and have a role in the expression of mucinous glycoproteins which protect the gut lumen.^1^^,^^2^
C. Ovarian cancer with peritoneal dissemination – Incorrect. Ovarian cancer can present with elevated CA-125 and peritoneal metastases; however, histological and immunohistochemical findings are inconsistent with ovarian cancer which would involve tumor cells in the dermis with nodular contours forming rudimentary ducts and intracytoplasmic inclusions and paired box 8, Wilms tumor-1, and estrogen receptor positivity.
D. Pancreatic cancer with liver metastasis – Incorrect. Pancreatic cancer would present with a different clinical picture with likely elevated CA-19-9. The biopsy findings and immunohistochemistry results do not align with pancreatic cancer which would demonstrate infiltration of the dermis by a ductal adenocarcinoma.
E. Primary peritoneal mesothelioma – Incorrect. Peritoneal mesothelioma uncommonly presents with gastrointestinal symptoms and is characterized by dermal proliferation of epithelioid cells with cytologic atypia that stain positive for Wilms tumor-1, mesothelin, podoplanin, and CK5/6.

**Question 2: Which of the following is a distinguishing factor in the dermatological presentation of Sister Mary Joseph nodules versus other cutaneous metastases? Sister Mary Joseph nodules are:**

A. Erythematous or violaceous tender paraumbilical or umbilical nodule
B. Multiple, well-circumscribed papules with central necrosis on the trunk
C. Violaceous plaques with a target-like center, predominantly on the back
D. Annular, hypopigmented plaques with peripheral scaling on the extremities
E. Discrete, nontender nodules with overlying telangiectasia on the face

**Answers:**

A. Erythematous or violaceous tender paraumbilical or umbilical nodule – Correct. A Sister Mary Joseph nodule is a metastatic lesion involving the umbilicus, manifesting as an indurated, firm colored nodule in an estimated 1% to 3% of cases where abdominopelvic malignancies metastasize to the umbilicus.^3^ Unlike the canonical appearance of a Sister Mary Joseph nodule, the patient described herein developed atypical rapid progressive skin changes (Fig 1). The presentation of cutaneous metastases can vary from multiple poorly circumscribed nodules to ulcerative plaques.
B. Multiple, well-circumscribed papules with central necrosis on the trunk – Incorrect. Well-circumscribed papules with central necrosis on the trunk are more characteristic of conditions like Kaposi’s sarcoma or certain infections, rather than Sister Mary Joseph nodules.
C. Violaceous plaques with a target-like center, predominantly on the back – Incorrect. These lesions would be indicative of conditions such as erythema multiforme or certain autoimmune disorders, not Sister Mary Joseph nodules.
D. Annular, hypopigmented plaques with peripheral scaling on the extremities – Incorrect. Such lesions are characteristic of conditions like tinea corporis or erythema annulare centrifugum, not Sister Mary Joseph nodules.
E. Discrete, nontender nodules with overlying telangiectasia on the face – Incorrect. These lesions are more characteristic of basal cell carcinoma or other cutaneous malignancies with a distinct vascular component.

**Question 3: What is the best treatment option for this patient’s skin findings?**

A. Surgical resection
B. Systemic chemotherapy
C. Intralesional chemotherapy
D. Topical chemotherapy
E. Supportive care

**Answers:**

A. Surgical resection – Incorrect. Although a standard gastrectomy is the primary surgical approach for locally advanced gastric signet ring cell adenocarcinoma, it is typically done with curative intent.^4^ Given the patient's advanced systemic disease, surgical intervention is less feasible and not likely curative.
B. Systemic chemotherapy – Correct. Systemic chemotherapy should be offered to patients with metastatic gastric signet ring cell adenocarcinoma for palliation and to improve survival.^5^ In cases of metastatic disease, a multimodal approach is frequently adopted, making effective oncology management crucial.
C. Intralesional chemotherapy – Incorrect. Intralesional therapies are designated for superficial lesions and are not suitable for systemic disease, which represents the primary underlying issue.
D. Topical chemotherapy – Incorrect. Similar to intralesional chemotherapy, topical chemotherapy is appropriate for superficial skin lesions but not systemic disease.
E. Supportive care – Incorrect. Supportive care alone would not be appropriate for managing metastatic cancer as a primary treatment option.

## Conflicts of interest

None disclosed.
